# DABCO-modified magnetic core-shell as an efficient nanocatalyst for synthesizing polyhydroquinoline derivatives

**DOI:** 10.3389/fchem.2025.1557628

**Published:** 2025-03-31

**Authors:** Mozhgan Esfandiari, Alireza Salimi Beni

**Affiliations:** Department of Chemistry, Faculty of Science, Yasouj University, Yasouj, Iran

**Keywords:** quaternary ammonium salt, magnetic properties, 1,4-diazabicyclo [2.2.2] octane (DABCO), heterogeneous nanocatalyst, hantzsch reaction, resorcinol-formaldehyde

## Abstract

The fabrication of core-shell structured magnetic resorcinol-formaldehyde composites has garnered considerable attention within the scientific community in recent years. A key area of focus has been the immobilization of homogeneous catalysts onto the surfaces of these materials and transforming them into heterogeneous catalysts. In this study, a novel quaternary ammonium salt catalyst was synthesized by immobilizing 1,4-diazabicyclo [2.2.2] octane (DABCO) on resorcinol-formaldehyde-modified Fe_3_O_4_ nanocomposite as a support (Fe_3_O_4_@RF/Pr-DABCO). The Fe_3_O_4_@RF/Pr-DABCO nanocomposite was characterized using various physicochemical techniques, including FT-IR, VSM, SEM, XRD, and TGA. The Fe_3_O_4_@RF/Pr-DABCO nanocomposite was employed as a power nanocatalyst in the Hantzsch reaction for synthesizing polyhydroquinoline derivatives using aromatic aldehydes, ammonium acetate, dimedone and ethyl acetoacetate. Various aromatic aldehydes were used as substrates in the presence of 0.003 g of Fe_3_O_4_@RF/Pr-DABCO under solvent-free condution at 60 °C, achieving high to excellent yields (90-99%) within short reaction times (5-15 min). Furthermore, this nanocatalyst showed excellent reusability and maintained its catalytic activity for at least eight consecutive cycles without a significant decrease in efficiency. These results demonstrate the potential of the Fe_3_O_4_@RF/Pr-DABCO nanocomposite as an efficient and sustainable catalyst for the synthesis of polyhydroquinoline derivatives via the Hantzsch reaction.

## 1 Introduction

Aligned with the principles of green chemistry, the development of recyclable and reusable catalytic systems has garnered significant interest among researchers over the past few decades. This focus on sustainable catalyst design aims to address environmental concerns and promote resource efficiency in chemical processes (Saberi et al., 2020). In recent years, the design of magnetic nanocatalysts is of great interest due to their very easy recovery using a simple magnet without the need for conventional and time-consuming filtration and reactions catalyzed by magnetic nanocatalysts have been widely studied due to the easy purification of products and efficient recovery of the catalyst (Shabir et al., 2020; Yazdani et al., 2025). Various magnetic nanoparticles (NPs) have been investigated for the development of magnetic catalytic systems. Among magnetic NPs, Fe_3_O_4_ NPs has been widely considered due to its low toxicity, easy and low-cost synthesis from cheap and readily available starting materials. Despite the promising attributes of Fe_3_O_4_ NPs, their practical applications are hindered by their high sensitivity to oxidation and aggregation, as well as their chemically reactive nature due to their high surface area (Serenjeh et al., 2015; Shaker and Elhamifar, 2020; Barzkar and Beni, 2023). These limitations necessitate the development of strategies to overcome these challenges and unlock the full potential of Fe_3_O_4_ NPs. One effective approach involves the creation of a suitable organic or inorganic coating on the surface of the magnetic NPs, which can enhance their stability and mitigate the aforementioned issues, thus paving the way for broader applications in various fields. Various materials have been utilized for coating magnetic nanoparticles, including silica, metal oxides, and polymers (Gong et al., 2018; Kilic et al., 2019; Shaker and Elhamifar, 2020; 2021b; Kilic et al., 2022). Notably, resorcinol-formaldehyde (RF) resin has emerged as a particularly promising option due to its exceptional properties. These properties encompass high stability, cost-effectiveness, facile structural manipulation, superior mechanical and thermal characteristics, impressive electrical conductivity, and a large surface area. The unique combination of these properties makes RF resin an attractive choice for coating magnetic nanoparticles, with the potential to enhance their performance and broaden their applications in diverse fields (Barzkar and Beni, 2020). Some of the recently developed reports include Fe_3_O_4_@RF-Au (Shi et al., 2024), Fe_3_O_4_@RF@void@PMO (Yu et al., 2019), Fe_3_O_4_@RF@void@PMO(IL)/Cu (Shaker and Elhamifar, 2021a), Fe_3_O_4_@SiO_2_@RF–SO_3_H (Barzkar and Beni, 2020) and Fe_3_O_4_@RF/Cu_2_O (Wang et al., 2017).

On the other hand, 1,4- Diazabicyclo [2.2.2] octane, also recognized as DABCO is known as a valuable ligand and catalyst for organic reactions due to its cost-effectiveness, environmental friendliness, non-toxicity, and high reactivity. However, recovering and removing DABCO in chemical reactions is complex. This problem has been solved by immobilizing DABCO on solid substrates, which can be easily filtered and reused multiple times (Baharfar and Azimi, 2014). Recent developments in this area, including Fe_3_O_4_@DT-Au (Kardan et al., 2022), Pd–DABCO@SiO_2_ (Jadhav et al., 2019), Ni(II)–DABCO@SiO_2_ (Hajipour and Abolfathi, 2017), Pd-DABCO-γ-Fe_2_O_3_ (Sobhani and Pakdin-Parizi, 2014), SB-DABCO (Hasaninejad et al., 2011) and Fe_3_O_4_@SiO_2_@DABCO (Gupta et al., 2023).

Meanwhile, heterocycles are a crucial and valuable group of chemical compounds, forming the basis of many pharmaceutical molecules and antibiotics. Currently, Synthesis of nitrogen-containing heterocycles, such as polyhydroquinoline and its derivatives, is important due to their medicinal properties. Polyhydroquinolines possess anti-diabetic, anti-cancer, anti-tumor, liver-protective, and skin-protective properties and treat cardiovascular diseases and Alzheimer’s (Shinde and Thakur, 2023). Polyhydroquinoline derivatives can be synthesized using the Hantzsch reaction, which has garnered significant attention. This reaction is a four-component reaction and is one of the first and most well-known multi-component reactions (Sajjadifar and Azmoudehfard, 2019; Shaker and Beni, 2021; Norouzi and Beiranvand, 2023). Due to the widespread use of polyhydroquinoline derivatives, various catalysts have been introduced for this reaction such as GuHCl (Baghbanian et al., 2010), ceric ammonium nitrate (CAN) (Ko and Yao, 2006), Sc(OTf)_3_ (Donelson et al., 2006), urease (Zhu and Li, 2021), palladium (0) nanoparticles (Saha and Pal, 2011), nickel nanoparticles (Sapkal et al., 2009), SBA-15@AMPD-Co (Ghorbani-Choghamarani et al., 2019), [βCD/Im](OTs)_2_ (Moheiseni et al., 2021), NiFe_2_O_4_MNPs (Ahankar et al., 2016), Fe_3_O_4_adenineNi (Tamoradi et al., 2018), CuSPATB/Fe_3_O_4_ (Ghorbani-Choghamarani et al., 2016), Fe_3_O_4_@SiO_2_PEG/NH_2_ (Kardooni et al., 2017) and Fe_3_O_4_ @SiO_2_@(CH_2_)_3_Im}C(NO_2_)_3_ (Yarie et al., 2016). Although most of the reports mentioned have obvious advantages, they also come with problems, such as toxic catalysts, volatile and harmful solvents, expensive metal precursors, high temperatures, the formation of by-products, and contamination of the final product. In most methods, the catalysts get damaged during the reaction and cannot be separated and reused. Therefore, developing efficient, recyclable, and environmentally friendly catalysts for synthesizing polyhydroquinolines is crucial (Kazemi and Mohammadi, 2020; Sharma et al., 2021).

**SCHEME 1:**
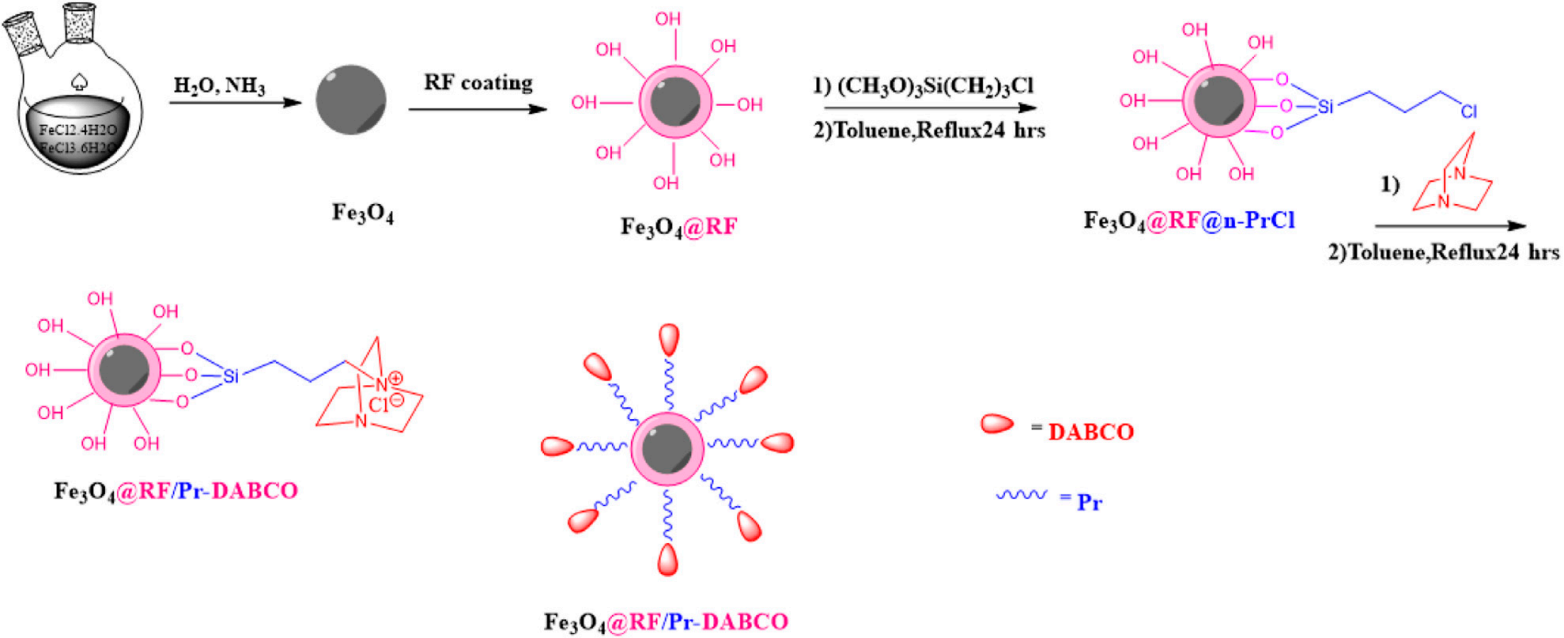
Preparation of Fe_3_O_4_@RF/Pr-DABCO nanocatalyst

In light of the significance of heterogeneous catalysis and the unique properties of 1,4-diazabicyclo [2.2.2]octane (DABCO), this study aims to design and synthesize a novel core-shell structured Fe_3_O_4_@RF/Pr-DABCO catalyst (Scheme 1). DABCO, selected for its crucial role as an organic group in preparing quaternary ammonium salt catalysts, can be readily immobilized on the Fe_3_O_4_@RF support. This approach offers the potential to create a highly efficient and recyclable catalytic system that capitalizes on the benefits of both the magnetic core and the catalytic activity of DABCO. Also, Fe_3_O_4_@RF/Pr-DABCO is applied as a powerful catalyst for synthesizing polyhydroquinoline derivatives under mild conditions.

## 2 Experimental section

### 2.1 Materials

All chemicals are used, such as formaldehyde solution (37 wt%), resorcinol, triethylamine, (3-aminopropyl)-trimethoxysilane, dimedone, ethyl acetoacetate, toluene dried, and ammonium acetate, were purchased from Merck, hydrochloric acid (37%) and Benzaldehyde and its derivatives were purchased from Sigma-Aldrich. The morphology of the particles was evaluated by TESCAN-Vega 3 scanning electron microscope (SEM) (Czech republic). Energy-dispersive X-ray spectroscopy (EDX) was obtained by using TESCAN-Vega 3 apparatus (Czechrepublic). Fourier transform infrared (FT-IR) spectroscopy was recorded on a PerkinElmer Spectrum2 spectrometer (United States). Thermal gravimetric analysis (TGA) and Differential Scanning (DSC) Calorimetry were performed using a SDT 650 (United States). Thermal gravimetric analysis (TGA) and differential thermal analysis (DTA) were performed using a STA6000 (United States). X-ray diffraction (XRD) was obtained using a Rigaku Ultima IV diffractometer (Japan). The magnetic properties of the particles were investigated using a vibrating sample magnetometer (VSM) of MDKB. (Iran). Melting points were determined using a KSB1N, Kruss apparatus in open capillary tubes (Germany). TLC-Grade-silica Gel-G/UV 254 thin layer chromatography (TLC) was used. A mixture of ethyl acetate and normal hexane was used for the mobile phase in the TLC tank. Ultrasonic model Elmasonic P60H was used to disperse the particles and to perform the organic reactions (Germany).

### 2.2 Synthesis of Fe_3_O_4_ nanoparticles

In a 100 mL round-bottom flask, 25 mL HCl (2 M), FeCl_3_.6H_2_O (5.2 g), and FeCl_2_.4H_2_O (2 g) were combined with a magnetic stirrer under argon atmosphere for 30 min at room temperature to form nanoparticles of Fe_3_O_4_ magnetite. Next, NH_4_OH (25%, 30 mL), was slowly injected into the solution over 30 min at 25°C. The obtained black precipitate was separated by a magnet and washed with water before drying in an oven (Kargar et al., 2024).

### 2.3 Synthesizing of Fe_3_O_4_@RF

In a 250 mL round-bottom flask, 1 g Fe_3_O_4_ particles were dispersed in water (20 mL) and ethanol (100 mL) using ultrasonic. Then (2 mL) of 25% ammonium hydroxide, formaldehyde (0.6 mL), and resorcinol (0.4 g) were added to the solution. The mixture was stirred for 10 h at 30°C under a magnetic stirrer and argon atmosphere, then was collected using a magnet and washed with water and ethanol three times, before finally drying at 60°C for 15 h (Mirbagheri et al., 2021).

### 2.4 Synthesis of Fe_3_O_4_@RF/PrCl

For this aim, in a 100 mL round-bottom flask, Fe_3_O_4_@RF nanoparticles (50 mg) were added to 50 mL of dry toluene and dispersed under ultrasonic waves. After that, 2 mL of (3-chloropropyl) trimethoxysilane was injected into the reaction. After refluxing under a magnetic stirrer and argon atmosphere for 24 h at 110°C, an external magnet filtered the product. The resultant product was washed with toluene and dried at 70°C for 15 h (Zare et al., 2020).

### 2.5 Synthesis of Fe_3_O_4_@RF/Pr-DABCO

In a 50 mL round-bottom flask, Fe_3_O_4_@RF/PrCl (1 g), DABCO (0.5 g), and triethylamine (0.4 mL) were dispersed under ultrasonic in 30 mL of dry toluene for 30 min. After 24 h of refluxing under a magnetic stirrer and argon atmosphere, the mixture was separated using a magnet, washed five times with dry toluene, and dried at 50°C for 5 h (Afsar et al., 2018).

### 2.6 Synthesis of polyhydroquinoline derivatives using Fe_3_O_4_@RF/Pr-DABCO

For this, aldehyde (1 mmol), ammonium acetate (1.4 mmol), dimedone (1 mmol), ethyl acetoacetate (1 mmol), and Fe_3_O_4_@RF/Pr-DABCO nanocatalyst (0.003 g) were added into a 5 mL round-bottom flask. The reaction mixture was located in an oil bath at 60°C, the temperature of which was previously adjusted by a heater-stirrer advice, and stirred under solvent-free condition and air atmosphere. The progress of the reaction was monitored by TLC (eluent; n-hexane: ethyl acetate, 4:6). After completion of the reaction, hot EtOH (10 mL) was added in the reaction vessel and catalyst was removed by an external magnet. Finally, the solvent was evaporated, and pure products resulted after recrystallization in EtOH (Nikoorazm and Erfani, 2019).

## 3 Results and discussion

### 3.1 Characterization of Fe_3_O_4_@RF/Pr-DABCO

**FIGURE 1:**
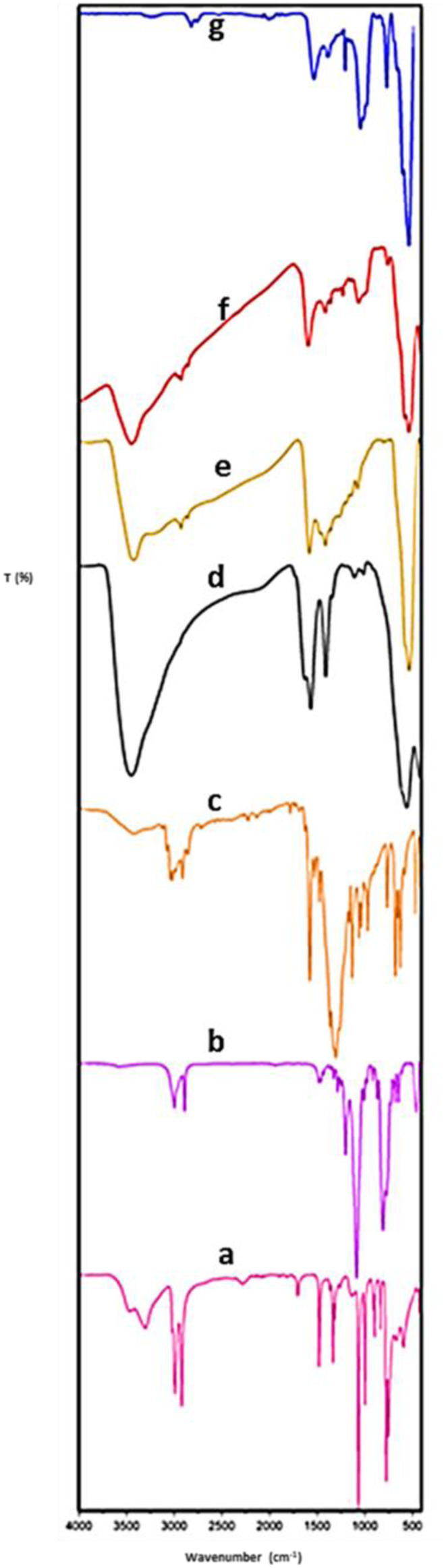
FT-IR spectrum of **(a)** DABCO, **(b)** Pr-Cl, **(c)** RF, **(d)** Fe_3_O_4_, **(e)** Fe_3_O_4_@RF, **(f)** Fe_3_O_4_@RF/Pr-Cl, **(g)** Fe_3_O_4_@RF/Pr-DABCO.

The FT-IR spectra of Fe_3_O_4_, RF, Fe_3_O_4_@RF, Pr-Cl, Fe_3_O_4_@RF/Pr-Cl, DABCO, and Fe_3_O_4_@RF/Pr-DABCO are shown in Figure 1. For Fe_3_O_4_, Fe_3_O_4_@RF, Fe_3_O_4_@RF/Pr-Cl and Fe_3_O_4_@RF/Pr-DABCO, the observed peak at 574 cm^−1^ is related to the stretching vibrations of the Fe-O bonds (Figures 1d–g). The FT-IR spectrum of Fe_3_O_4_@ RF (Figure 1e) exhibits peaks at 3,010, 2,854–2,974 cm^−1^attributable to the vibrations of C-H aromatic and CH_2_ moieties of RF resin in Fe_3_O_4_@RF, respectively. Also, the absorption bands at 1,615 and 1,453 cm^−1^ correspond to the aromatic rings in the RF. These results are in perfect agreement with the RF spectrum (Figure 1c), thus confirming the successful formation of RF resin on the Fe_3_O_4_ surface. (Pol et al., 2014; Barzkar and Beni, 2020). The FT-IR spectrum of Fe_3_O_4_@RF/Pr-Cl (Figure 1f) shows the C-H stretching vibration at 2,950 and 2,826 cm^−1^, the C–Cl absorption band at 800 cm^−1^, and Si-O stretching at 1,000–1,100 cm^−1^, which perfectly match with the observed peaks in the pure Pr-Cl spectrum (Figure 1b). These results prove the successful formation of the Fe_3_O_4_@RF/Pr-Cl composite (Hamidinasab et al., 2020; Honari et al., 2022). FT-IR pure DABCO (Figure 1a) exhibits a peak at 1,250 cm^−1^ (C–N stretching), 1,475 cm^−1^ (CH_2_ bending), and 2,850–2,964 cm^−1^ (C-H stretching vibrations) (Tafti et al., 2024). Thus, for the Fe_3_O_4_@RF/Pr-DABCO, the presence of a peak at 1,250 cm^−1^ (C–N stretching) proved immobilization of DABCO groups on the of Fe_3_O_4_@RF/Pr-Cl composite (Figure 1g) (Hasaninejad et al., 2011; Nasseri and Sadeghzadeh, 2014).

**FIGURE 2:**
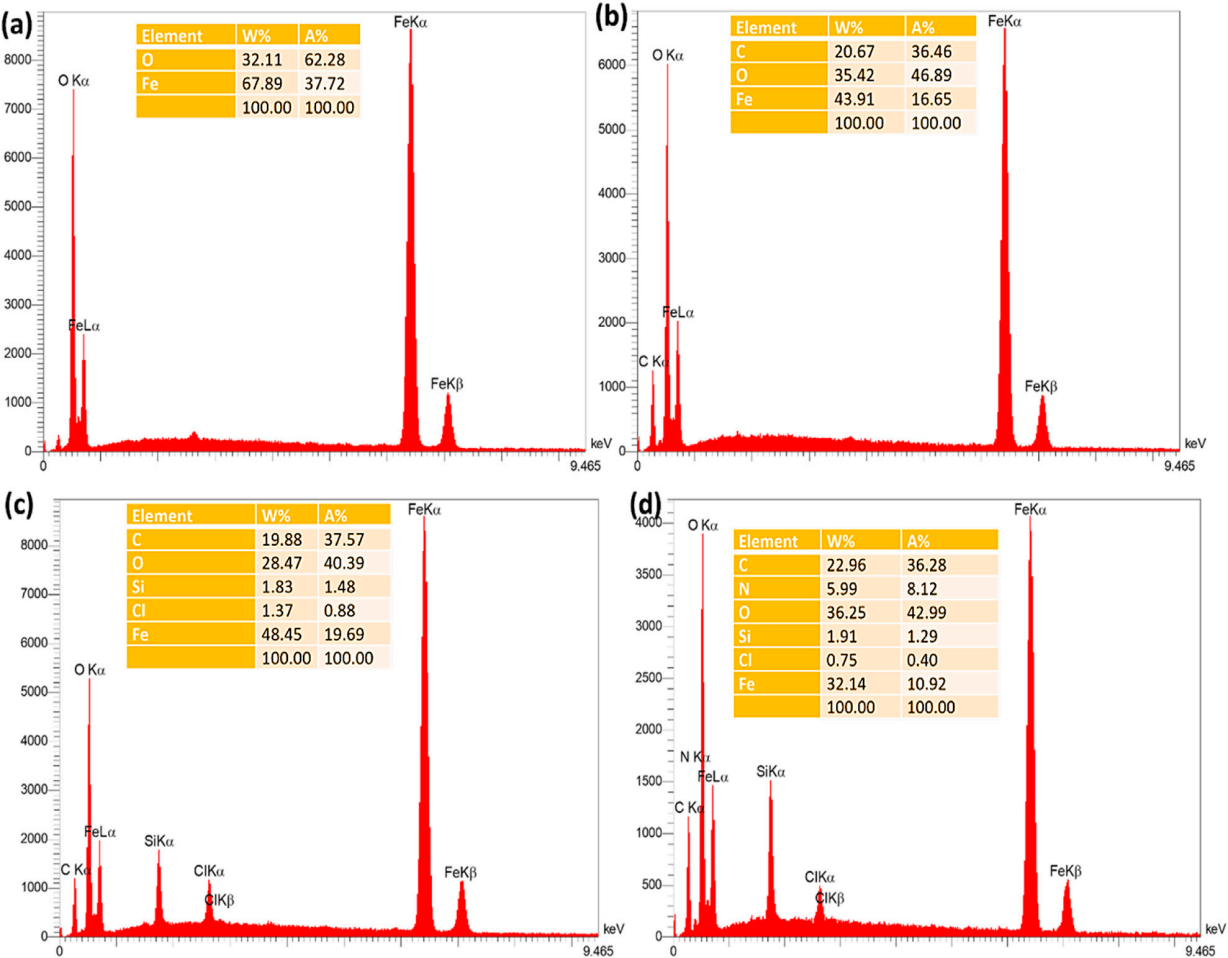
EDX spectrum of the **(a)** Fe_3_O_4_, **(b)** Fe_3_O_4_@RF, **(c)** Fe_3_O_4_@RF/Pr-Cl, and **(d)** Fe_3_O_4_@RF/Pr-DABCO.

**FIGURE 3:**
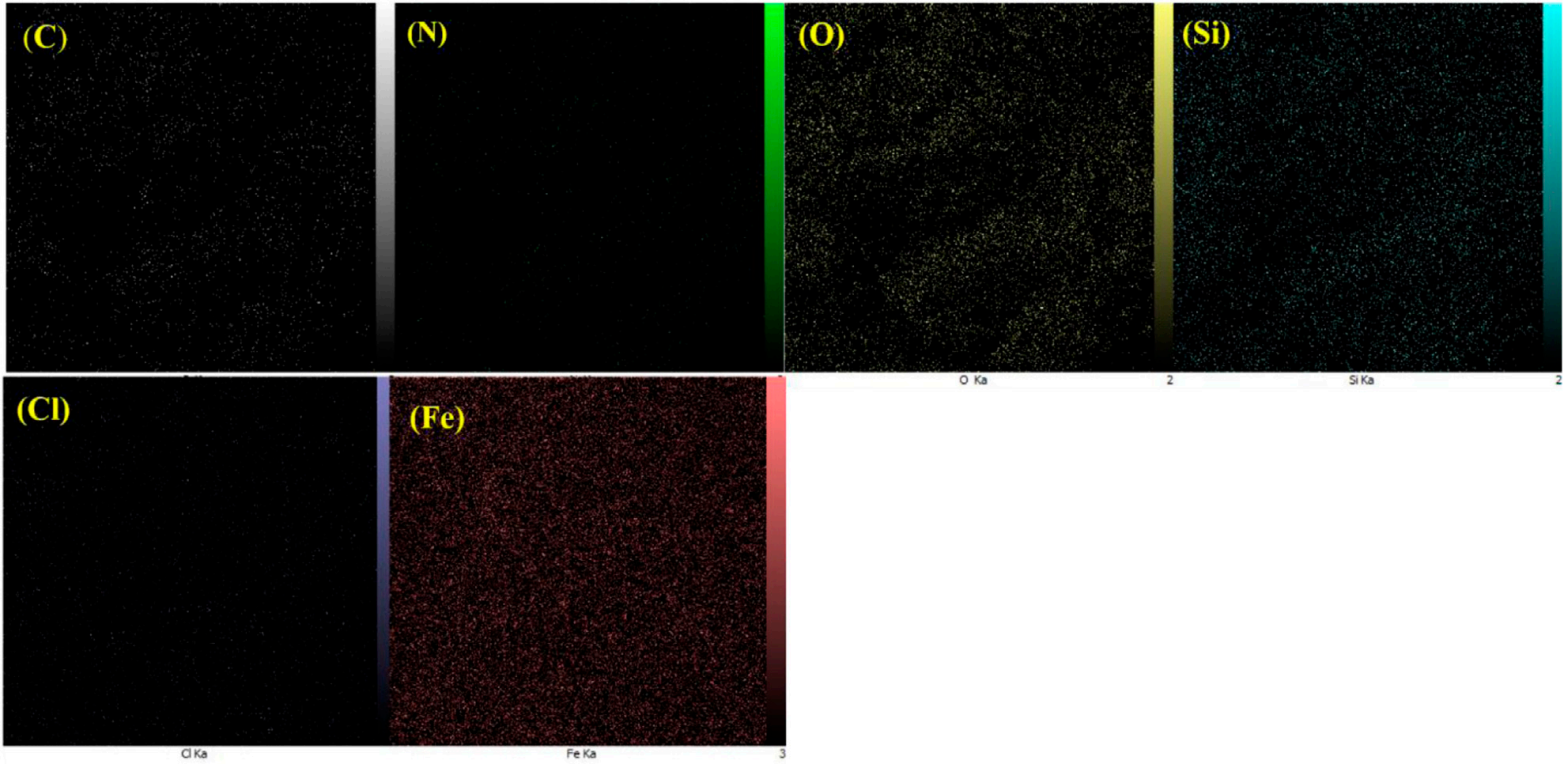
Element mapping of the Fe_3_O_4_@RF/Pr-DABCO nanocatalyst.

The EDX spectrum of Fe_3_O_4_ nanoparticles demonstrated the presence of Fe and O elements, proving the successful production of these nanoparticles (Figure 2a). To confirm the successful modification of the Fe_3_O_4_ surface with RF resin, the Fe_3_O_4_@RF material was also characterized by EDX analysis. The appearance of C-signal in the latter analysis confirms successful-production of RF resin on Fe_3_O_4_ surface (Figure 2b). Furthermore, to prove the successful chemical immobilization of (3-chloropropyl) trimethoxysilane on Fe_3_O_4_@RF nanocomposite, the Fe_3_O_4_@RF/PrCl nanocomposite was also characterized by EDX analysis. The advent of Si and Cl elements proving successful-immobilization of (3-chloropropyl) trimethoxysilane species on Fe_3_O_4_@RF nanocomposite (Figure 2c). Finally, to confirm the successful immobilization of DABCO on Fe_3_O_4_@RF/PrCl nanocomposite, the final material was characterized by EDX and the appearance of N-signal confirmed immobilization of DABCO species (Figure 2d). The EDX mapping analysis was also done to display the dispensation of elements in the Fe_3_O_4_@RF/Pr-DABCO framework. This analysis demonstrated that all elements are uniformly distributed in the Fe_3_O_4_@RF/Pr-DABCO framework (Figure 3).

**FIGURE 4:**
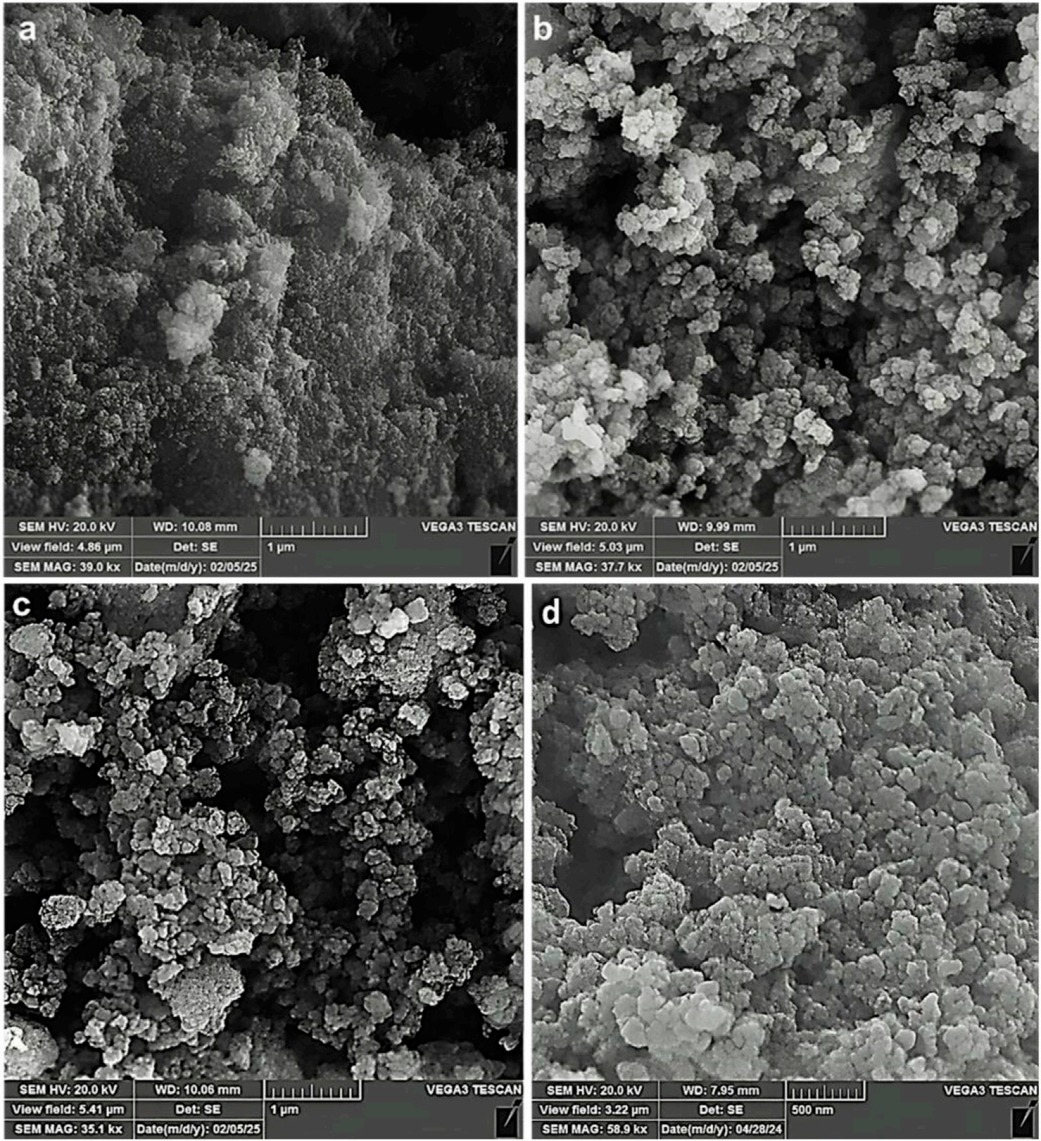
SEM image of the **(a)** Fe_3_O_4_, **(b)** Fe_3_O_4_@RF, **(c)** Fe_3_O_4_@RF/Pr-Cl, and **(d)** Fe_3_O_4_@RF/Pr-DABCO.

The morphology of the particles at different steps of nanocomposite preparation was investigated by using SEM (Figure 4). The SEM analysis revealed a consistent spherical morphology with uniform particle size distribution at different stages of the process. Additionally, it was observed that the size of the nanoparticles (NPs) increased incrementally at each step compared to the previous stage.

**FIGURE 5:**
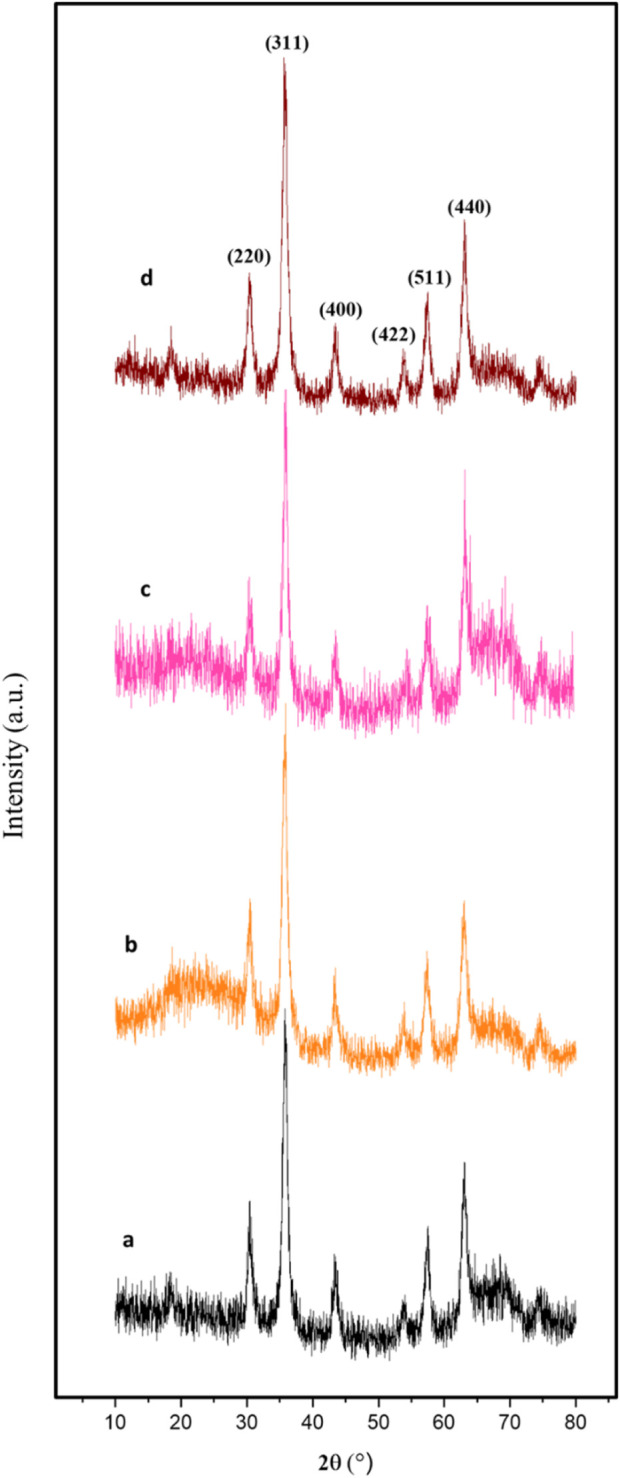
XRD pattern of the **(a)** Fe_3_O_4_, **(b)** Fe_3_O_4_@RF, **(c)** Fe_3_O_4_@RF/Pr-Cl, and **(d)** Fe_3_O_4_@RF/Pr-DABCO.

The powder X-ray diffraction (PXRD) analysis of Fe_3_O_4_, Fe_3_O_4_@RF, Fe_3_O_4_@RF/Pr-Cl, and Fe_3_O_4_@RF/Pr-DABCO nanomaterials showed six sharp peaks at 2θ = 30.3, 35.6, 43.4, 53.7, 57.4, and 63.1°, corresponding to Miller indices of 220, 311, 400, 422, 511, and 440, respectively. The results of this analysis prove that the Fe_3_O_4_ crystalline structure is preserved during the synthesizing processes of Fe_3_O_4_@RF/Pr-DABCO nanocamposite (Figure 5) (Hashkavayi and Raoof, 2017).

**FIGURE 6:**
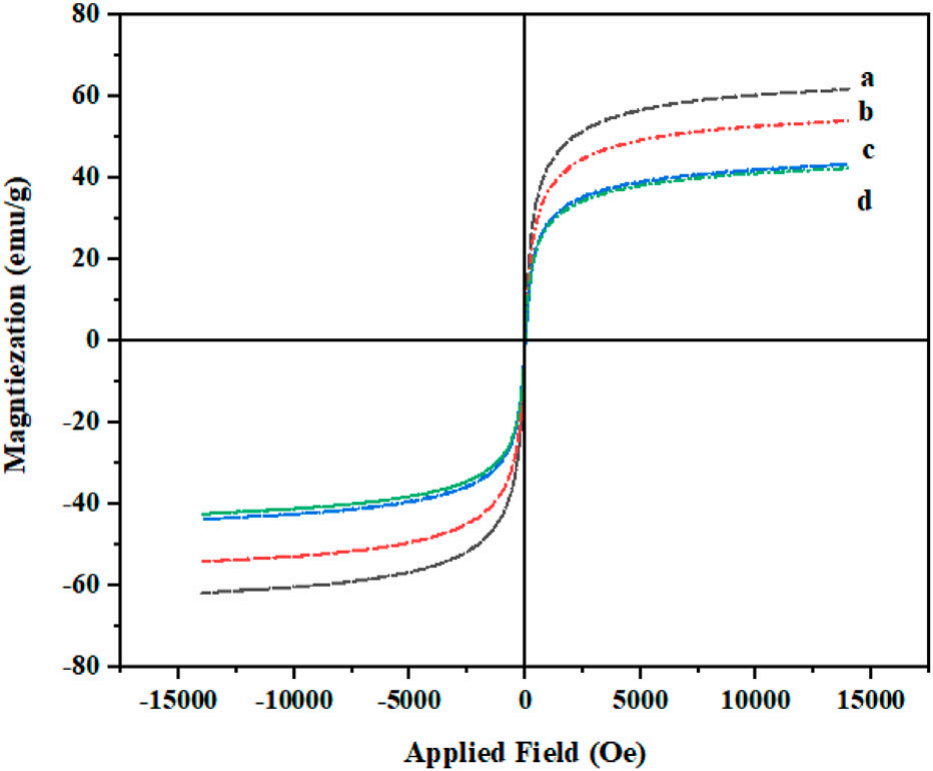
VSM (a) Fe_3_O_4_, (b) Fe_3_O_4_@RF, (c) Fe_3_O_4_@RF/Pr-Cl, and (d) Fe_3_O_4_@RF/Pr-DABCO.

The magnetic properties of Fe_3_O_4_, Fe_3_O_4_@RF, Fe3O4@RF, Fe_3_O_4_@RF/Pr-Cl, and Fe_3_O_4_@RF/Pr-DABCO nanomaterials were investigated by VSM \analysis at room temperature (Figure 6). The results of this analysis demonstrated that all samples have a superparamagnetic behavior. The magnetic saturation of Fe_3_O_4_, Fe_3_O_4_@RF, Fe_3_O_4_@RF/Pr-Cl, and Fe_3_O_4_@RF/Pr-DABCO nanomaterials were 61.98, 53.87, 42.23, and 41.96 emu/g, respectively. The decrease in saturation magnetization, after each step, confirms successful chemical immobilization of Resorcinol-Formaldehyde precursors and DABCO moieties on the surface of the Fe_3_O_4_ NPs. However, the magnetic property of Fe_3_O_4_@RF/Pr-DABCO is still sufficient, and it can be easily recovered using an external magnet.

**FIGURE 7:**
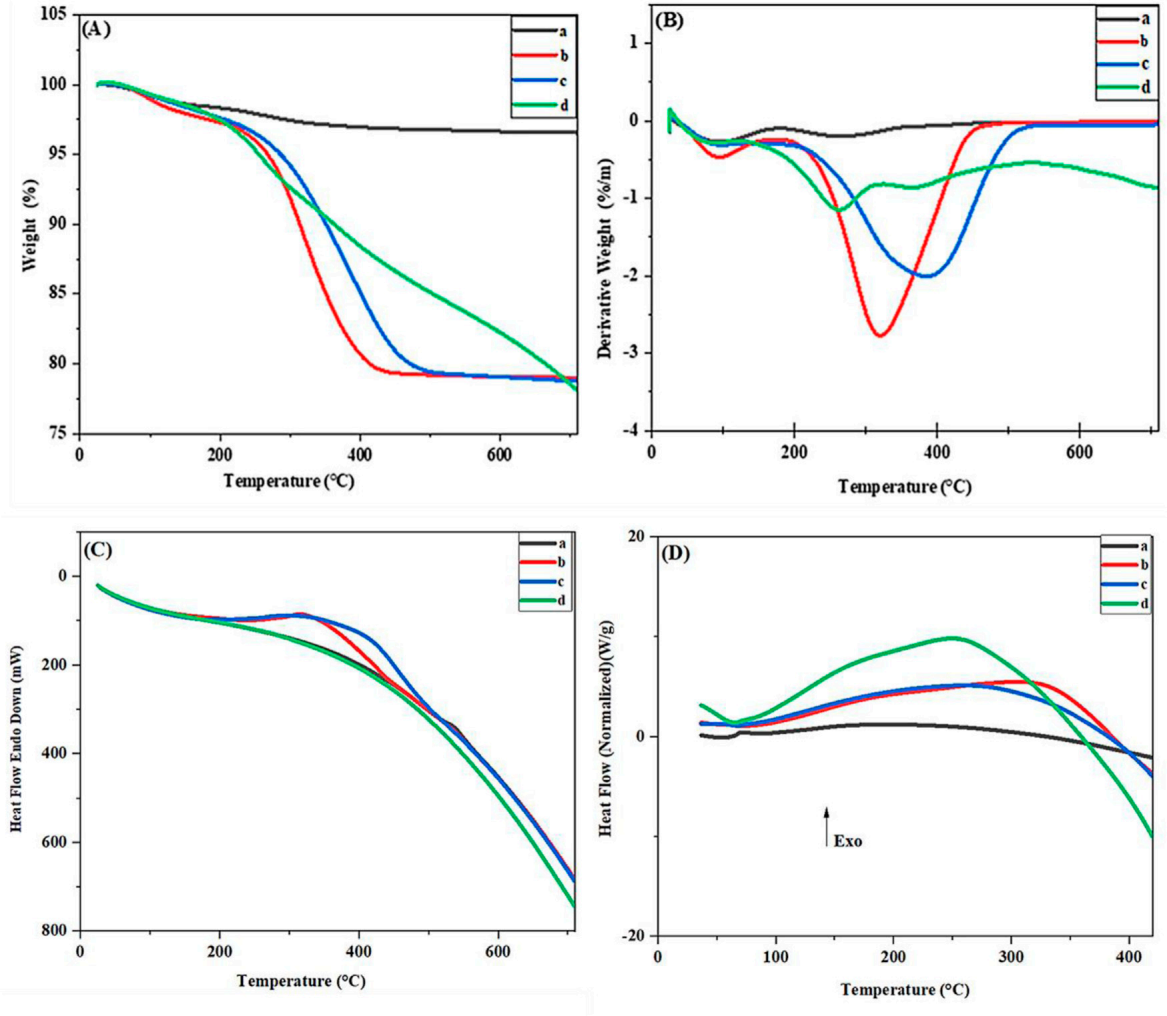
**(A)** TGA **(B)** DTG, **(C)** DTA, and **(D)** DSC of the **(A)** Fe_3_O_4_, **(B)** Fe_3_O_4_@RF, **(C)** Fe_3_O_4_@RF/Pr-Cl, and **(D)** Fe_3_O_4_@RF/Pr-DABCO.

To further investigate the compositional structure and thermal stability, thermogravimetric analysis (TGA), derivative thermogravimetry (DTG), differential thermal analysis (DTA), and Differential Scanning Calorimetry (DSC) were performed at different stages of nanocatalyst synthesis (Figures 7A–D). The TGA diagram of Fe_3_O_4_ (Figure 7Aa) shows two mass loss stages. The first stage, with a mass loss of 1.66%, at temperatures between 25°C and 200°C, is related to water evaporation, and the second stage, with a mass loss of 1.96%, at temperatures between 200°C and 600°C, is connected to the decomposition of hydroxyl groups on the Fe_3_O_4_ surface., the final mass loss is 3.62% (Zhang et al., 2021). The mass loss graph of Fe_3_O_4_@RF (Figure 7Ab) shows two stages of mass loss. In the first stage, at temperatures below 200°C, due to the evaporation of water or residual physically adsorbed solvent, the observed mass loss is 2.55%, While the second mass loss15.13% between 200°C and 469°C is related to the decomposition of the RF shell, the final mass loss is 17.68% (Zheng et al., 2016). The mass loss graph of Fe_3_O_4_@RF/Pr-Cl (Figures 7Ac) has two stages of mass loss. The first mass loss of about 2.49% is related to the residual other solvents or water on the surface of magnetic nanoparticles, which appears below 200°C. The main weight loss, with a mass loss of 18.59%, from 200°C to 600°C, is due to the decomposition of the chloropropyl group and RF shell; the final mass loss is 21.08% (Dehghani et al., 2025). TGA of Fe_3_O_4_@RF/Pr-DABCO shows two distinct stages of mass loss (Figures 7Ad). The first stage, with an onset of degradation at 50°C–200°C, with mass loss of 1.85%is caused by the evaporation of H_2_O or residual solvent on the catalyst’s surface. The second stage, between 200°C and 700°C, with mass loss of 20.78%, is related to continuous decomposition of the organic components (DABCO, propyl group and RF shell). The final mass loss is 22.63% (Rajabi-Salek et al., 2018; Jadidi Nejad et al., 2020; Jia et al., 2022).

TGA and DTG (Figure 7B) data were supported by DTA and DSC curves. Based on DTA and DSC (Figures 7C, D), the separation process of organic materials attached to the surface of Fe_3_O_4_@RF/Pr-DABCO nanocatalyst is exothermic, and the different intensity of the peaks indicates the synthesis of different stages of the nanocatalyst.

Comparison of the mass loss curve and thermal stability (TGA) graph analysis of each step of the synthesis of the Fe_3_O_4_@RF/Pr-DABCO magnetic nanocatalyst confirmed that all four steps of the nanocatalyst synthesis were successfully completed and the alkyl group and DABCO was loaded onto the surface of the Fe_3_O_4_@RF particles. Also, the TGA thermograms of the product obtained at each stage of synthesis are parallel to the elemental composition obtained by EDX.

### 3.2 Catalytic activity of the Fe_3_O_4_@RF/Pr-DABCO

Following the successful characterization of Fe_3_O_4_@RF/Pr-DABCO, the effectiveness of the catalyst was assessed in the green production of polyhydroquinoline. The condensation between 1.4 mmol of NH_4_OAc, 1 mmol of benzaldehyde, 1 mmol of ethyl acetoacetate, and 1 mmol of dimedone was chosen as a model reaction. The findings indicate that the catalyst, solvent, and temperature significantly affect the reaction’s progression. Consequently, the amount of catalyst, choice of solvent, and temperature were evaluated to enhance the reaction conditions.

Initially, to evaluate the effectiveness of the Fe_3_O_4_@RF/Pr-DABCO catalyst, it was observed that the reaction did not occur in the absence of the catalyst, indicating its essential role in the process (Table 1, entry 1). To further examine the catalyst’s impact, we tested various amounts of the catalyst (1, 3, 5, 7, and 9 mg). As detailed in Table 1 entries 2–6, the reaction yield improved with increased catalyst quantity from 0.001 g to 0.003 g (Table 1, entry 3). The optimal yield was achieved with 0.003 g of the catalyst (Table 1, entry 3). Notably, a catalyst amount of 0.005 g produced the same yield as 0.003 g (Table 1, entry 4). Additionally, increasing the catalyst amount from 0.007 to 0.009 g resulted in a minor reduction in product yield. Studies have shown that excessive amounts of catalyst alter the reaction mechanism and increase side reactions, leading to the formation of unwanted by-products. This change reduces the selectivity towards the desired product and consequently reduces its yield (Chen et al., 2021; Platero et al., 2022).

**TABLE 1:** The reaction conditions optimized for the synthesis of polyhydroquinolines by Fe_3_O_4_@RF/Pr-DABCO

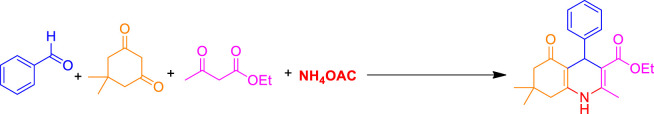						
Entry	Catalyst	Catalyst amount (g)	Solvent	T (°C)	T (min)	Yield (%)^b^ ^,^ ^c^
1	Fe_3_O_4_@RF/Pr-DABCO	None	—	60	150	---
2	Fe_3_O_4_@RF/Pr-DABCO	0.001 g	solvent-free	60	15	69
3 ^a^	Fe_3_O_4_@RF/Pr-DABCO	0.003 g	solvent-free	60	15	97
4	Fe_3_O_4_@RF/Pr-DABCO	0.005 g	solvent-free	60	15	97
5	Fe_3_O_4_@RF/Pr-DABCO	0.007 g	solvent-free	60	15	93
6	Fe_3_O_4_@RF/Pr-DABCO	0.009 g	solvent-free	60	15	90
7	Fe_3_O_4_@RF/Pr-DABCO	0.003 g	solvent-free	25	15	27
8	Fe_3_O_4_@RF/Pr-DABCO	0.003 g	solvent-free	40	15	77
9	Fe_3_O_4_@RF/Pr-DABCO	0.003 g	solvent-free	70	15	96
10	Fe_3_O_4_@RF/Pr-DABCO	0.003 g	solvent-free	90	15	89
11	Fe_3_O_4_@RF/Pr-DABCO	0.003 g	DMSO	60	15	66
12	Fe_3_O_4_@RF/Pr-DABCO	0.003 g	Toluene	60	15	40
13	Fe_3_O_4_@RF/Pr-DABCO	0.003 g	H_2_O	60	15	70
14	Fe_3_O_4_@RF/Pr-DABCO	0.003 g	CH_3_CN	60	15	66
15	Fe_3_O_4_@RF/Pr-DABCO	0.003 g	EtOH	60	15	80
16	Fe_3_O_4_	0.003	solvent-free	60	15	64
17	Fe_3_O_4_@RF	0.003	solvent-free	60	15	Trace
18	Fe_3_O_4_@RF/PrCl	0.003	solvent-free	60	15	Trace

^a^ Bold values indicate the optimum condition.

^b^ Isolated yields.

^c^ Molecular weight of aromatic aldehyde ≡ molecular weight of product x g of aromatic aldehyde ≡ y g.

$\text{Theoretical yield}\left(y\right)=\frac{\text{molecular}\;\text{weight}\;\text{of}\;\text{product}\times x\;g\;\text{of}\;\text{aromatic}\;\text{aldehyde}}{\text{molecular}\;\text{weight}\;\text{of}\;\text{aromatic}\;\text{aldehyde}}$

$\text{Isolated yield}=\frac{\text{Actual}\;\text{yield}}{\;\text{Theoretical}\;\text{yield}}\times 100$ (Dhengale et al., 2021).

The model reaction was subsequently evaluated at various temperatures, specifically 25°C, 40°C, 60°C, 70°C, and 90°C (Table 1, entry 3 vs. entries 7–10). An increase in temperature from 25°C to 60°C resulted in enhanced reaction efficiency, with the optimal yield observed at 60°C (Table 1, entry 3). Additionally, satisfactory efficiency was noted at 70°C (Table 1, entry 9). However, as the temperature rose to 90°C, there was a decline in product yield (Table 1, entry 10). Studies have shown that by-products are formed at 90°C, and therefore the yield of the main product is reduced.

This study examined the impact of various solvents, such as dimethyl sulfoxide, toluene, water, acetonitrile, and ethanol, as well as solvent-free conditions (Table 1, entry 3 vs. entries 11–15). The findings indicated that the highest yield achieved was 97% under solvent-free conditions. Because DABCO is an organic compound and the solvents are all organic, the interaction between DABCO and the solvents increases, and the accumulation of substrate around the catalytic active site decreases, and the catalytic activity of DABCO decreases compared to solvent-free conditions. In the presence of ethanol and water solvents, although these solvents also interact with DABCO and reduce its catalytic activity, these solvents can have hydrogen interactions with the substrates, causing substrate activation and facilitating the reaction conditions. However, in general, solvent-free conditions are the best option for this process and do not reduce catalytic activity (Lu et al., 2012; Varghese and Mushrif, 2019).

To ascertain the pivotal role of DABCO groups in the catalytic cycle, the efficacy of DABCO-free Fe_3_O_4_, Fe_3_O_4_@RF, and Fe_3_O_4_@RF/PrCl nanomaterials was investigated under identical reaction conditions and time as the Fe_3_O_4_@RF/Pr-DABCO catalyst (Table 1, entry 3 vs. entries 16–18). Notably, the latter catalysts exhibited negligible product yields, unequivocally establishing the indispensable presence of DABCO species as crucial catalytic centers for the reaction.

A diverse range of polyhydroquinolines was synthesized under optimal conditions, utilizing 0.003 g of a solvent-free catalyst at 60°C with Fe_3_O_4_@RF/Pr-DABCO as a heterogeneous catalyst. As detailed in Table 2, various benzaldehydes featuring both electron-donating and electron-withdrawing groups were employed in the synthesis of polyhydroquinolines, resulting in products characterized by high yields, brief reaction times, and precise melting point measurements (Table 2, entries 1–7).

**TABLE 2:** Synthesis of polyhydroquinolines utilizing Fe_3_O_4_@RF/Pr-DABCO under ideal conditionsa

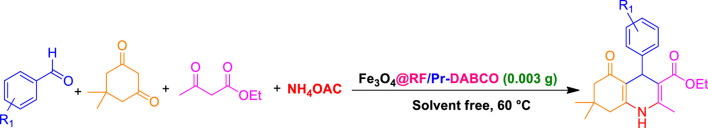						
Entry	R1	Product	T (min)	Yield (%)b	M.P. °C	(Ref.)
1	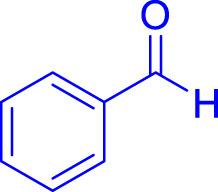	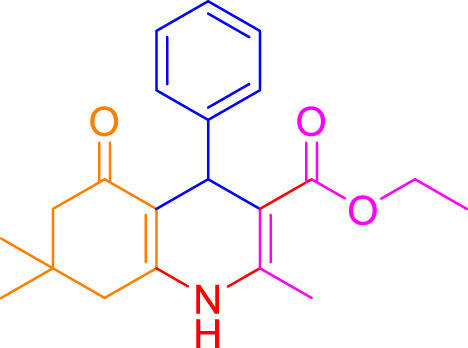	15	97	203–207	Elhamifar et al. (2017)
2	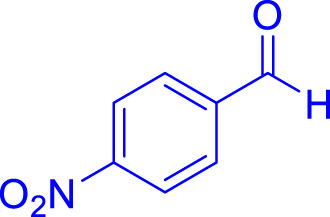	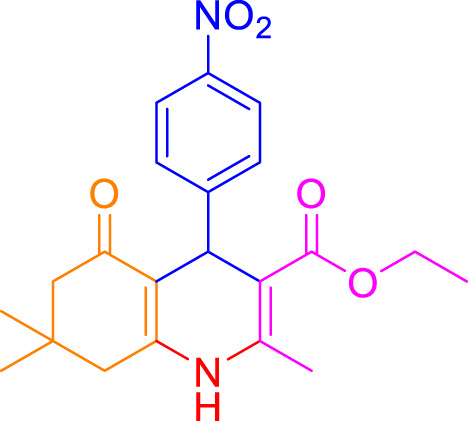	15	90	245–246	Kumar et al. (2008)
3	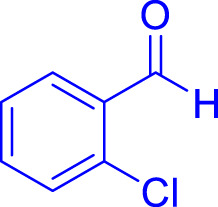	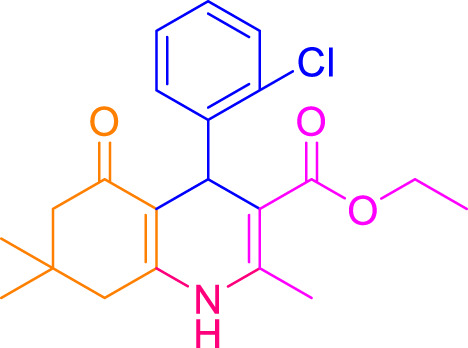	10	94	206–208	Elhamifar and Ardeshirfard (2017)
4	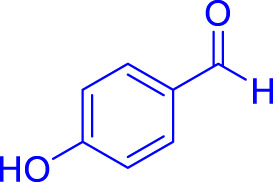	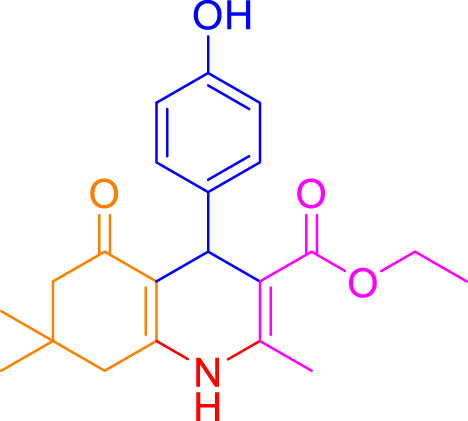	10	94	238–240	Khazaei et al. (2014)
5	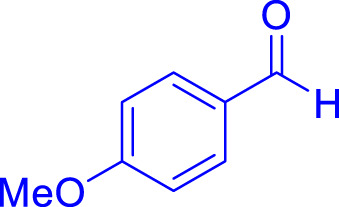	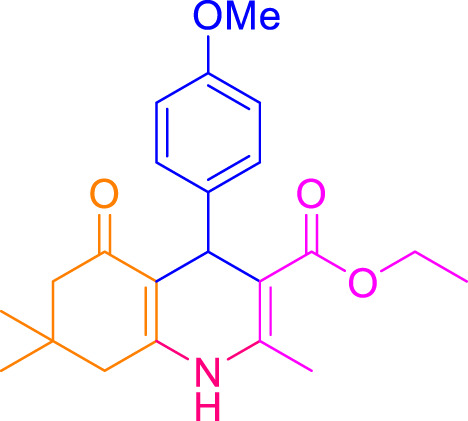	10	99	256–257	Surasani et al. (2012)
6	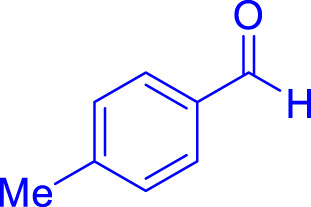	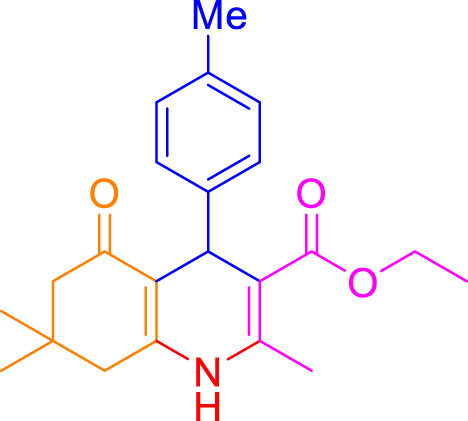	10	97	260–262	Cherkupally and Mekala (2008)
7	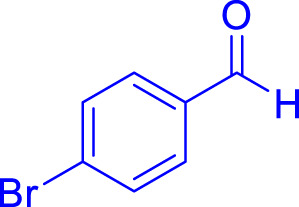	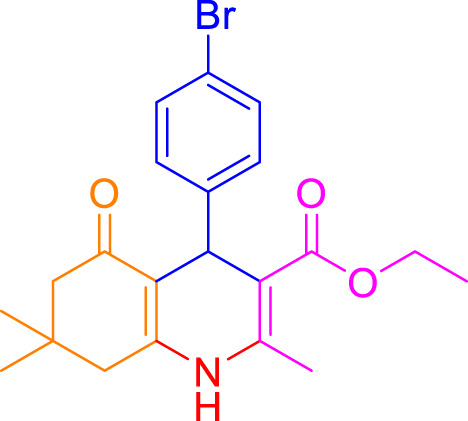	5	99	254–256	Wang et al. (2005)

^a^ Reaction parameters: at 60°C, ammonium acetate (1.4 mmol), dimedone (1 mmol), ethyl acetoacetate (1 mmol), aldehyde (1 mmol), and catalyst (0.003 g) without solvent.

^b^ Isolated yields.

**FIGURE 8:**
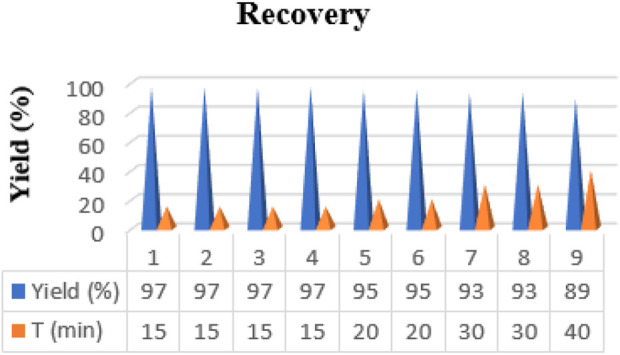
Reusability of the Fe_3_O_4_@RF/Pr-DABCO

The recovery and reusability of the catalyst were tested for eco-friendly industrial and commercial applications. A test model evaluated the condensation of 0.003 mg of the catalyst with ethyl acetoacetate (1 mmol), dimedone (1 mmol) benzaldehyde (1 mmol), and NH_4_OAc (1.4 mmol). The catalyst was isolated from the solution following each run through an external magnet, washed with ethanol, dried, and then used again in another reaction to regenerate it. According to Figure 8, the recycling process can be conducted under identical conditions to the initial run at least eight times without experiencing any substantial loss.

**FIGURE 9:**
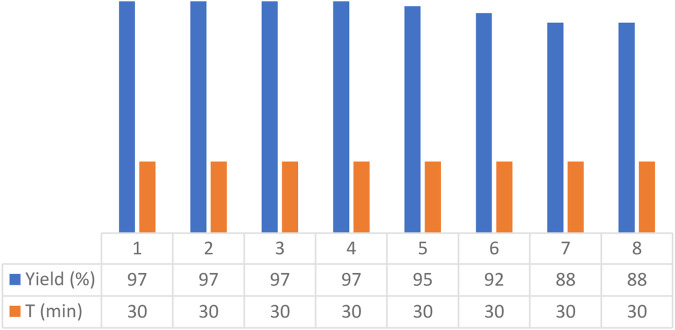
Recoverability and reusability of the Fe_3_O_4_@RF/Pr-DABCO at a fixed time.

In another experiment, the recoverability and reusability of the Fe_3_O_4_@RF/Pr-DABCO catalyst were investigated at a fixed time under optimal conditions. In this test, the reaction time for each run was 30 min. Following each run, the catalyst was separated using a magnetic field and subsequently reused in the next run, under the same conditions as the first run. As illustrated in Figure 9, this catalyst can be recovered and reused at least 8 times without a substantial decrease in its performance.

**SCHEME 2:**
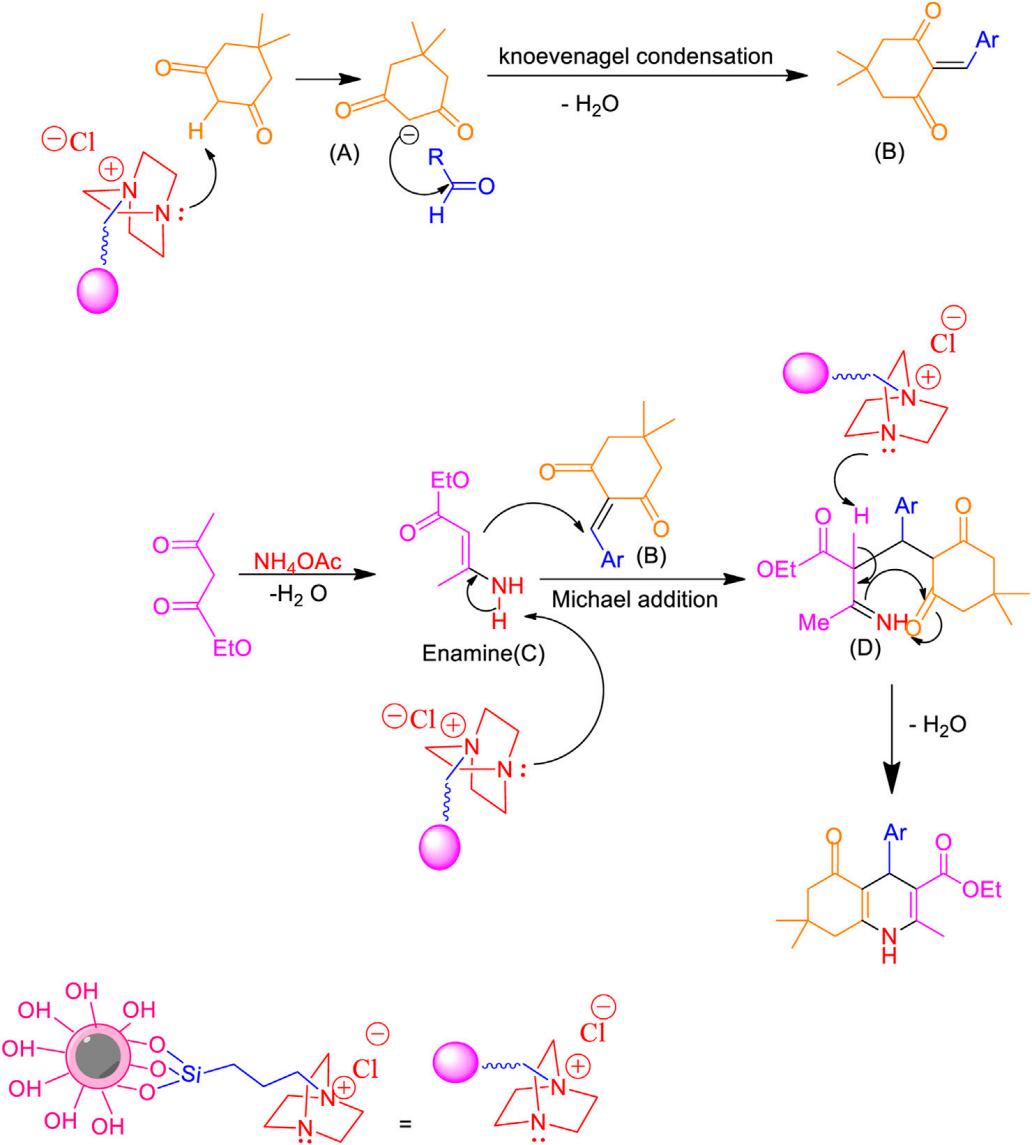
A suitable catalytic mechanism for the production of polyhydroquinolines using Fe_3_O_4_@RF/Pr-DABCO

Scheme 2 presents a proposed mechanism for synthesizing polyhydroquinolines using the Fe_3_O_4_@RF/Pr-DABCO as a catalyst. Initially, the carbanion group (A) is generated through the deprotonation of the α-proton of dimedone by the basic nitrogen group of the catalyst, DABCO. Subsequently, intermediate (B) is formed via a Knoevenagel condensation reaction between carbanion (A) and an aldehyde. The formation of intermediate (C) occurs after the elimination of a water molecule from the reaction involving ammonia (derived from ammonium acetate) and ethyl acetoacetate. The Michael addition of intermediate (B) with (C) results in the formation of intermediate (D). Ultimately, the nanocatalyst facilitates the preparation of the final product by promoting the cyclization of (D) and the removal of a water molecule (Al Anazi et al., 2023).

We investigated the catalytic performance of magnetic Fe_3_O_4_@RF/Pr-DABCO in producing polyhydroquinoline derivatives and compared it to previously reported nanocatalysts (Table 3). The results show that our designed catalyst is cost-effective, easy to use, and highly efficient, yielding high amounts of polyhydroquinoline derivatives under typical reaction conditions. Therefore, this catalyst is comparable to or even better than previous catalysts in Hantzsch’s synthesis of polyhydroquinolines in terms of reaction conditions, yield, and ease of magnetic recovery.

**TABLE 3:** Compare the effectiveness of the current catalyst with other catalysts.

Entry	Catalyst	Conditions	Yield (%)	Ref.
1	SBA-Pr-SO_3_H	Cat. 0.05g, solvent-free, 80°C., 10 min	85	Singh et al. (2018)
2	Sulfated polyborate	Cat 80 mg, Solvent free,100°C, 20 min	93	Aute et al. (2020b)
3	Fe_3_O_4_@SiO_2_/ZnCl_2_	Cat 5 mg, Solvent free, 110°C, 30 min	92	Maleki et al. (2019)
4	Aluminized polyborate	Cat 0.075, Solvent free, 100°C, 15 min	94	Aute et al. (2020a)
5	Ru^III^@CMC/Fe_3_O_4_	Cat 0.5 mol%, Solvent free,80°C,20 min	92	Chen et al. (2019)
6	Fe_3_O_4_@RF/Pr-DABCO	Cat 0.003g, Solvent-free,60°C., 15 min	97	This work

## 4 Conclusion

In summary, a novel magnetic RF modified with DABCO (Fe_3_O_4_@RF/Pr-DABCO) was successfully synthesized. The EDX and FT-IR analyses confirmed successful chemical immobilization of resorcinol-formaldehyde precursors and DABCO moieties on the surface of the Fe_3_O_4_ NPs. Also, TGA analysis proved the good immobilization of resorcinol-formaldehyde resin and DABCO moieties onto Fe_3_O_4_ NPs and showed the high thermal stability of the Fe_3_O_4_@RF/Pr-DABCO nanocomposite. The SEM image showed that the Fe_3_O_4_@RF/Pr-DABCO nanostructures are spherical and regular. Also, The XRD demonstrated the structure of Fe3O4 is not changed under the conditions of the synthesis of Fe_3_O_4_@RF/Pr-DABCO nanocomposite. Finally, its catalytic application was studied as a catalyst for synthesizing polyhydroquinoline derivatives. This nanocatalyst, showed excellent catalytic activity with a minimum amount (0.003 g) at an optimum temperature of 60°C in only 15 min and effectively led to the formation of products with high yields (97%–99%). Additionally, the Fe_3_O_4_@RF/Pr-DABCO nanocatalyst can be easily recovered using an external magnet and could be reused up to eight times without any significant loss of its catalytic activity.

## Data Availability

The original contributions presented in the study are included in the article/supplementary material, further inquiries can be directed to the corresponding authors.
